# Principles of artificial intelligence in radiooncology

**DOI:** 10.1007/s00066-024-02272-0

**Published:** 2024-08-06

**Authors:** Yixing Huang, Ahmed Gomaa, Daniel Höfler, Philipp Schubert, Udo Gaipl, Benjamin Frey, Rainer Fietkau, Christoph Bert, Florian Putz

**Affiliations:** 1 Department of Radiation Oncology, Universitätsklinikum Erlangen, Friedrich-Alexander Universität Erlangen-Nürnberg, 91054 Erlangen, Germany; 2 Comprehensive Cancer Center Erlangen-EMN (CCC ER-EMN), 91054 Erlangen, Germany; 3 Translational Radiobiology, Department of Radiation Oncology, Universitätsklinikum Erlangen, Friedrich-Alexander Universität Erlangen-Nürnberg, 91054 Erlangen, Germany

**Keywords:** Radiation oncology, Artificial intelligence, Machine learning, Deep learning, Tutorial

## Abstract

**Purpose:**

In the rapidly expanding field of artificial intelligence (AI) there is a wealth of literature detailing the myriad applications of AI, particularly in the realm of deep learning. However, a review that elucidates the technical principles of deep learning as relevant to radiation oncology in an easily understandable manner is still notably lacking. This paper aims to fill this gap by providing a comprehensive guide to the principles of deep learning that is specifically tailored toward radiation oncology.

**Methods:**

In light of the extensive variety of AI methodologies, this review selectively concentrates on the specific domain of deep learning. It emphasizes the principal categories of deep learning models and delineates the methodologies for training these models effectively.

**Results:**

This review initially delineates the distinctions between AI and deep learning as well as between supervised and unsupervised learning. Subsequently, it elucidates the fundamental principles of major deep learning models, encompassing multilayer perceptrons (MLPs), convolutional neural networks (CNNs), recurrent neural networks (RNNs), transformers, generative adversarial networks (GANs), diffusion-based generative models, and reinforcement learning. For each category, it presents representative networks alongside their specific applications in radiation oncology. Moreover, the review outlines critical factors essential for training deep learning models, such as data preprocessing, loss functions, optimizers, and other pivotal training parameters including learning rate and batch size.

**Conclusion:**

This review provides a comprehensive overview of deep learning principles tailored toward radiation oncology. It aims to enhance the understanding of AI-based research and software applications, thereby bridging the gap between complex technological concepts and clinical practice in radiation oncology.

## Introduction

Radiation therapy stands as one of the cornerstones of the multidisciplinary management of cancer, harnessing targeted ionizing radiation to eradicate malignant cells while sparing surrounding healthy tissue. As with many other fields in medicine, the advent and rapid evolution of artificial intelligence (AI) technologies, in particular deep learning, promise to revolutionize the landscape of radiation oncology, thereby fostering improvements in treatment planning, accuracy, personalization, and patient outcomes [1, 2]. AI’s potential in radiation oncology is vast, from delineating tumors to optimizing radiation dosage, predicting responses, and monitoring potential side effects [3]. However, for modern-day radiooncologists and medical physics experts, navigating the complexities of AI principles, methodologies, and applications can be daunting. This review endeavors to bridge this knowledge gap, providing a comprehensive and accessible guide to the principles of AI as they pertain to radiation therapy. By understanding the intersection of AI with radiation oncology, practitioners can be better positioned to harness these technologies for improved patient care in the future.

In the rapidly expanding world of AI, there exists a wealth of literature detailing intricate deep learning techniques and their myriad applications [4, 5]. While these comprehensive resources offer in-depth perspectives, they are often tailored to audiences with a strong foundation in machine learning and computational sciences. Many such publications can be labyrinthine for clinicians and medical professionals, potentially hindering the effective assimilation of this knowledge into clinical practice. Recognizing this gap, the present review has been meticulously crafted with radiooncologists and medical physics experts in mind. Our aim is to demystify complex AI methodologies, thus offering a digestible and clinically relevant overview that allows for the seamless integration of AI into the realm of radiation therapy. This guide stands as a bridge between the sophisticated world of deep learning and the practical necessities of the radiooncology clinic.

## General principles

### Artificial intelligence, machine learning, deep learning, and radiomics

**Fig. 1:**
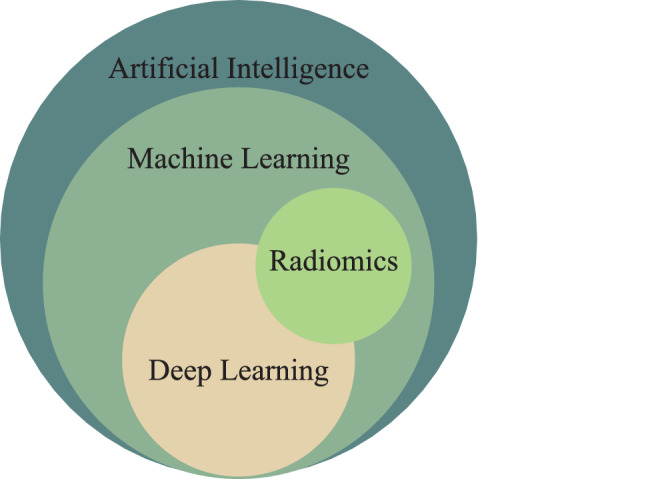
Relationships between artificial intelligence, machine learning, deep learning, and radiomics. Radiomics can use conventional machine learning or latest deep learning techniques to extract features from medical images

The relationships between AI, machine learning, deep learning, and radiomics are displayed in Fig. 1. AI includes everything from simple, rule-based algorithms to complex, learning-based systems. A program which can sense, reason, act, and adapt can be regarded as AI. Machine learning represents a subset of AI technologies. It refers to the ability of machines to learn from data. Deep learning represents a subset of machine learning techniques. It involves neural networks with many layers (hence “deep”) that can learn from data. Radiomics [6] is a process to extract computational imaging features of any kind from medical images with the purpose of predicting medical endpoints such as treatment effectiveness and potential side effects [7], which has become a well-known concept in radiation oncology. The conventional concept of radiomics refers to the specific classical workflow using predefined features such as PyRadiomics features [8] and the conventional classification and regression algorithms like support vector machines (SVMs) [9]. Since comparable imaging features can be extracted by convolutional neural networks (CNNs) via convolution [10], the broader concept of radiomics has nowadays extended from conventional machine learning into deep learning, as illustrated in Fig. 1.

### General components of training a deep learning model

In deep learning, the training process is essential for developing models that can make accurate predictions or decisions based on data. This process involves several key components: epochs, batches, learning rate, optimizers, loss functions, overfitting, and validation, as well as separate datasets for training, validation, and testing. An epoch represents a full pass through the entire training dataset, where the model updates its weights. Training data are often divided into smaller subsets called batches, which are processed sequentially during an epoch. The learning rate determines the step size at each iteration while moving toward a minimum of the loss function, influencing how quickly a model learns. Optimizers [11] are algorithms or methods used to change the attributes of the neural network, such as weights and learning rate, to reduce the losses. A loss function measures how well the model’s predictions correspond to the actual values, and minimizing this loss is crucial for learning from the data. Overfitting occurs when a model learns not just the underlying pattern but also the noise in the training data, leading to poor performance on unseen data. Techniques like regularization are used to prevent this, and the model’s performance is continually assessed on a validation dataset to tune parameters and select the best model iteration. After training and validation, the model is evaluated on a test dataset, providing a final measure of the model’s performance in real-world scenarios. Effective management of these components enhances the model’s ability to learn accurately and predict reliably.

### Supervised vs. unsupervised learning

Supervised and unsupervised learning are two foundational paradigms in machine learning. The key distinction between supervised and unsupervised learning is whether labels are used for training the AI model. Supervised learning involves training models on labeled datasets, whereby both the input data and the corresponding desired outputs are provided. For example, to train a brain metastasis segmentation model, input medical images and their corresponding metastasis segmentation labels are necessary during the training phase [12]. In contrast, unsupervised learning does not require explicit labels for training. Instead, it delves into the inner features of the data, aiming to unveil hidden structures or patterns. Examples include clustering patients into groups based on similar tumor profiles to understand disease subtypes [13] or using dimensionality-reduction techniques to represent complex patient data in a more interpretable manner [14].

Fundamentally, the distinguishment between supervised and unsupervised learning relies on the loss/objective function: if the loss function definition requires a label, it is supervised learning; otherwise, it is unsupervised learning. For example, training a brain metastasis segmentation model [12] can use the following binary cross entropy (BCE) loss function:

1 $$\mathcal{L}_{ \textrm{BCE}}(p,y)=-(y\times\log(p)+(1-y)\times\log(1-p)),$$

where $p$ is the network output probability of each pixel being a metastasis and $y$ is its corresponding classification label (1 for metastasis and 0 for normal tissue). Hence, it is supervised learning. Instead, training a network to reduce noise in computed tomography (CT) images [15] with the following total variation (TV) loss is an example of unsupervised learning:

2 $$\mathcal{L}_{ \textrm{TV}}(\boldsymbol{f})=\sum_{i}||\nabla\boldsymbol{f}_{i}||_{2},$$

where $\boldsymbol{f}$ is the input image and $i$ is the pixel index. The nabla operator $\nabla$ is a gradient operator which calculates the pixel intensity differences between the $i-$th pixel and its neighboring pixels. The $\ell_{2}$ norm $||\cdot||_{2}$ represents the magnitude of the change. The definition of TV is the sum of all absolute changes in pixel intensity. TV employs the intrinsic sparsity nature of clean medical images [16] and, hence, no additional labels are required.

Another example of unsupervised learning is using an autoencoder network [17, 18] to reconstruct its input. Autoencoder networks have ample applications and can be used for image-denoising applications (like CT/cone-beam CT or MRI improvement in radiotherapy) [19] but also for abnormality detection (e.g., in radiotherapy plans) [20] or for deriving deep radiomics features from image data among other things [21]. The $\mathcal{L}_{2}$ loss can be used for training the autoencoder:

3 $$\mathcal{L}_{2}(\boldsymbol{f})=||\mathcal{M}(\boldsymbol{f})-\boldsymbol{f}||_{2},$$

where $\mathcal{M}$ is the autoencoder model and $\mathcal{M}(\boldsymbol{f})$ is the model output with the input image $\boldsymbol{f}$. As the autoencoder aims to restore the original input image as accurately as possible, no additional labels are required.

**Fig. 2:**
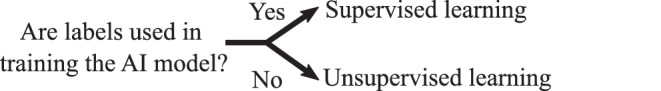
The key distinction between supervised and unsupervised learning is whether labels are used for training the AI model

### Model explainability

Deep learning models, particularly those involving complex architectures like deep neural networks, are often regarded as “black boxes” due to their intricate structures and the opaqueness of their decision-making processes. To address this, many methods have been developed to enhance their explainability. Feature importance methods such as Shapley Additive exPlanations (SHAP) [22] and the feature importance ranking measure (FIRM) [23] determine the importance of each feature in the decision-making process. Feature visualization methods like saliency maps [24] and gradient-weighted class activation mapping (Grad-CAM) [25] provide a heat map in the input image to highlight the input regions which are most relevant to the model’s decision. Transformer-based networks [26] with multihead self-attention mechanisms reveal the important visual or textual regions intrinsically by the attention weights. Please refer to survey papers of [27, 28] for such model explainability methods.

The above methods try to interpret models with complex architectures. Another direction is to build networks based on known mathematical or physical operators. Such networks are known as physics-informed neural networks (PINNs) [29, 30]. Since such networks use data-driven methods to improve the precision of conventional methods, the concept is also called “precision learning” [31]. For example, networks can be built based on the mathematical reaction–diffusion equations for glioma growth modeling [32, 33].

## Types of deep learning models

In the field of radiation oncology, recent advancements have been significantly driven by deep learning algorithms, which represent the forefront of modern AI applications. Consequently, the following section introduces various types of deep learning models.

### Multilayer perceptron (MLP)

**Fig. 3:**
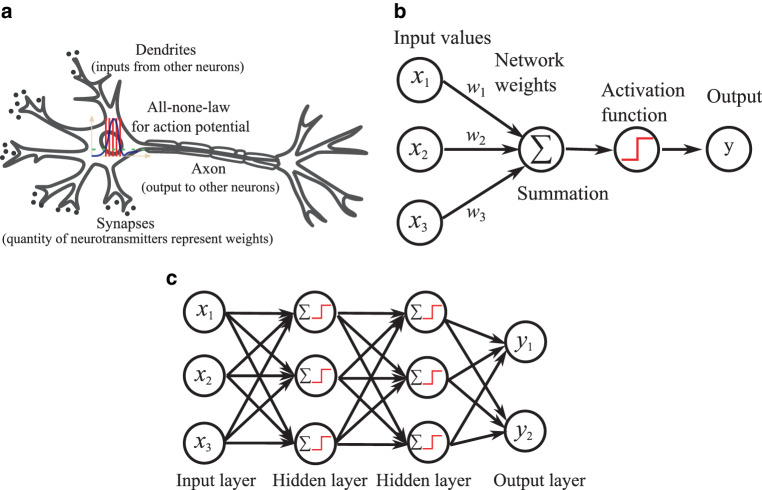
Illustration of a perceptron and an MLP. A perceptron applies a nonlinear activation to the weighted sum of input feature values, where the ReLU activation function is used as an example. An MLP consists of multiple layers and each hidden layer consists of multiple perceptrons. **a** Perceptron, **b** Perceptron, **c** MLP

Before the advent of convolutional neural networks (CNNs) [34], artificial neural networks mainly referred to multilayer perceptrons (MLP), which are also called fully connected neural networks. The basic component of an MLP is called a perceptron [35], which resembles a biological neuron (Fig. 3a) in human or animal nervous systems. A perceptron applies a nonlinear activation (resembling the nonlinear all-or-none principle of a biological neuron, in which the magnitude of the action potential is independent of the magnitude of the input stimulus as long as the threshold is reached) to the weighted sum or combination of input feature values (corresponding to the dendrites integrating the synaptic input in a biological neuron). Therefore, mathematically, a perceptron (“artificial neuron”) simply corresponds to *a linear regression followed by a nonlinear activation*, as displayed in Fig. 3b. In radiation oncology, linear regression is commonly used:

4 $$\bar{y}=w_{0}+w_{1}x_{1}+w_{2}x_{2}+{\ldots}+w_{k}x_{k}=\boldsymbol{w}\cdot\boldsymbol{x},$$

where $$\bar{y}$$ is the linear regression output, $$\boldsymbol{x}^{\top}=[1,x_{1},x_{2},{\ldots},]$$$$x_{k}]$$ is the feature vector (superscript $$\top$$ denotes the transpose of $$\boldsymbol{x}$$), $$\boldsymbol{w}=[w_{0},w_{1},w_{2},{\ldots},w_{k}]$$ is the weight vector, and $$\boldsymbol{w}\cdot\boldsymbol{x}$$ is the vector dot product of both vectors. By convention and throughout this review, bold lower-case symbols (e.g., $$\boldsymbol{w}$$) refer to vectors, bold upper-case symbols (e.g., $$\boldsymbol{W}$$) refer to matrices, and non-bold italic symbols refer to scalar values. Note that the bias $$w_{0}$$ has already been merged into the weight vector $$\boldsymbol{w}$$. A perceptron is hence mathematically defined as

5 $$y=\delta(\bar{y})=\delta(\boldsymbol{w}\cdot\boldsymbol{x}),$$

where $\delta$ is a nonlinear activation function. Here, only one output value $y$ is generated. To generate multiple output values as a vector $\boldsymbol{y}$, different weight vectors can be stacked row by row to form a weight matrix $\boldsymbol{W}$, i.e.,

6 $$\boldsymbol{y}=\delta(\bar{\boldsymbol{y}})=\delta(\boldsymbol{W}\cdot\boldsymbol{x}).$$

In a perceptron, various types of nonlinear activation functions can be used. These functions are crucial because they introduce nonlinearity into the model, enabling the network to learn more complex relationships in the data. Some commonly used nonlinear functions in perceptrons include step function (defined as $\delta(x)=( \textrm{sign}(x)+1)/2$), sigmoid/logistic function ($\delta(x)=\frac{1}{1+e^{-x}}$) [36], hyperbolic tangent function (tanh; $\delta(x)=\frac{e^{x}-e^{-x}}{e^{x}+e^{-x}}$) [37], rectified linear unit (ReLU; $\delta(x)=\max(0,x)$) [38], leaky ReLU ($\delta(x)=\max(x,\alpha x)$, where $\alpha$ is a small positive number such as 0.01) [39], and the swish function ($\delta(x)=x\cdot\frac{1}{1+e^{-\beta x}}$, where the scaling parameter $\beta$ can be learned) [40]. Such functions are also widely employed in modern networks, with the ReLU function being the most frequently used due to its piecewise linearity [41], which has advantages for network optimization and interpretability.

An MLP consists of an input layer, an output layer, and multiple hidden layers. Each hidden layer consists of multiple perceptrons. The universal approximation theorem [42] states that theoretically, an MLP with a single, infinitely wide hidden layer can approximate arbitrary functions, which is the theoretical foundation of AI. In practice, however, an infinitely wide hidden layer is impossible. Hence, increasing layer depth or number is a practical way to increase network representation power [43], which has empirically proven to be extremely effective. Increasing the number of layers enabled new levels of network performance and novel applications that were previously unachievable with shallower network architectures. Because of this, the term “deep” in “deep learning” mainly refers to the network depth. One intuitive explanation for the increased performance with increasing network depth is that increasing the number of layers allows the network to learn features at different levels of abstraction.

### Convolutional neural networks (CNNs)

#### General principles of CNNs

**Fig. 4:**
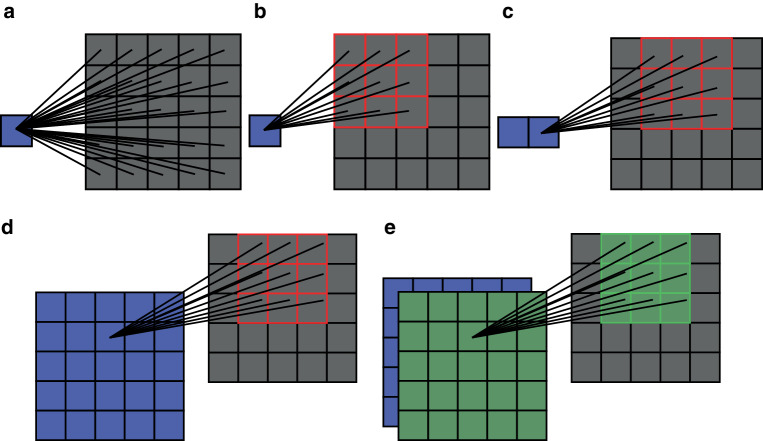
The numbers of weights required to obtain a feature value from an image differ between multilayer perceptrons (MLPs) and convolutional neural networks (CNNs): **a** 25 weights are required to calculate the feature value for a $5\times 5$ image in an MLP; **b** 9 weights are required to calculate the feature value in a $3\times 3$ convolution kernel (the *red grid*); **c** the same 9 weights are used to get new feature values by moving the convolution kernel position within the image; **d** the complete $5\times 5$ feature map generated by the same convolution kernel from an input image (with zero padding); **e** multiple feature maps can be generated by multiple convolution kernels/filters, which form different feature channels. **a** A perceptron in an MLP, **b** A perceptron in a CNN, **c** The next perceptron in a CNN, **d** A feature map generated by one convolution kernel in a CNN, **e** Multiple feature maps generated by multiple convolution kernels in a CNN

CNNs have risen as a pivotal cornerstone [34], especially when dealing with image data. Like MLPs, we call the basic component of CNNs a convolutional perceptron. It also applies a nonlinear activation function to the weighted combination of input feature values. The difference is that a convolutional perceptron sums up a neighborhood of input values which is determined by the convolution kernel size or window (Fig. 4b), whereas a perceptron in MLPs sums up all the input values as a fully connected node (Fig. 4a). Fundamentally, a convolutional perceptron is the same as a regular perceptron, but with the weights set to 0 for input values outside the convolution window. Another key difference is that the same convolutional perceptron with the same weights is used to generate the next output value by simply moving the convolution kernel position within the image, as displayed in Fig. 4c, whereas a new perceptron with different weights is necessary for an MLP. In the example of Fig. 4, to generate one output feature from a $5\times 5$ image requires training of 25 weights and, hence, generation of a $5\times 5$ feature map requires $25\times 25$ weights for an MLP. In stark contrast, for a CNN, only 9 weights must be trained to generate a $5\times 5$ feature map (Fig. 4d. The number of weights differs drastically for medical images with a size of $512\times 512$, where an MLP requires $512^{4}$ weights to generate a $512\times 512$ feature map, but a CNN requires the same 9 weights to generate a $512\times 512$ feature map. Therefore, CNNs are more efficient for image processing because they reduce the number of parameters (from $512^{4}$ to 9 for one feature map in this example) through weight sharing. For network training, too many parameters in a model relative to the quantity of available training data will cause overfitting. Because of the lower number of parameters, CNNs are less prone to overfitting than MLPs.

**Fig. 5:**
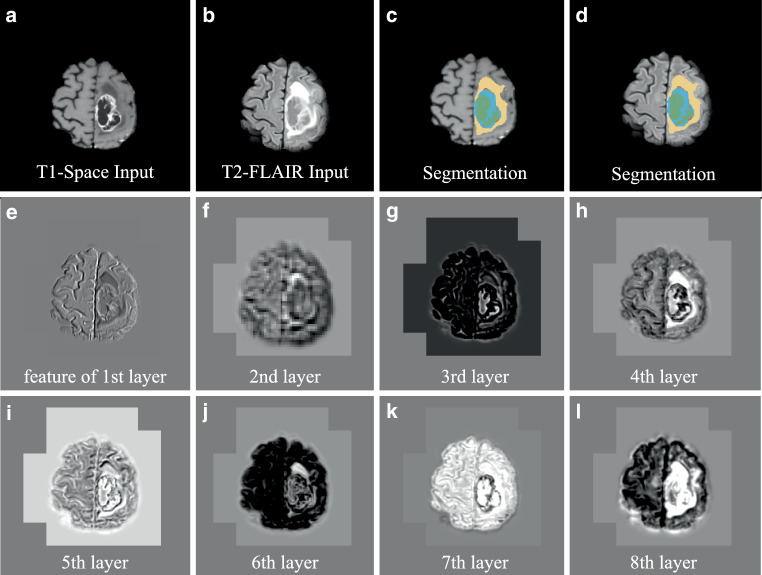
Some exemplary features extracted by DeepMedic [44] for glioma segmentation. The colors in the segmentation represent different tumor subregions: edema/invasion (*yellow*), nonenhancing tumor (*brown*), enhancing core (*cyan*), and necrotic core (*green*); **a** and **b** are T1-space and T2-FLAIR input images, respectively; **c** and **d** are the network segmentations overlaid with **a** and **b**, respectively; **e**–**l** are the first-channel features of the 1st to 8th convolutional layers in the first pathway of DeepMedic (displayed in an intensity window of $[-1,1]$)

Characterized by their unique convolutional layers, CNNs are adept at automatically extracting hierarchical features from images. Since one convolution kernel can only extract limited features, a convolutional layer typically uses multiple kernels to generate multiple feature maps, as displayed in Fig. 4e, which form different feature channels for subsequent convolutions. A deep CNN typically consists of tens of convolutional layers, which initially extract low-level features such as edges and progressively discern highly abstract features in the deeper layers [45–47]. https://arxiv.org/pdf/1311.2901.pdf (Fig. 2) in [45] provides a good visual illustration of extracted features in different layers of a CNN for computer vision tasks. Fig. 5 in the current work exemplifies the feature maps of different layers in the DeepMedic [44] network for glioma segmentation. Such automatic spatial feature extraction is particularly invaluable for tasks in radiooncology imaging like image enhancement [48–50], organ segmentation [51–53], and others [1, 54].

#### Representative networks of CNN

**Fig. 6:**
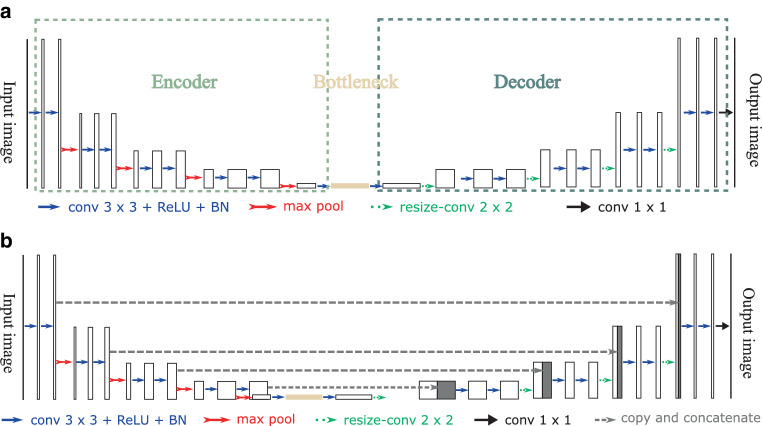
Architectures of a convolutional autoencoder and a U-Net. A convolutional autoencoder consists of an encoder, a bottleneck (latent representation), and a decoder, where convolutional layers are used to extract features. The U‑Net can be regarded as a modified version of a convolutional autoencoder, where features from the encoder are *copied and concatenated *to the decoder at different feature levels to employ multilevel features more effectively. **a** A convolutional autoencoder, **b** A U-Net

**Convolutional autoencoders** Autoencoders [17, 18] are commonly used to compress high-dimensional data into a low dimensional latent representation. An autoencoder consists of an encoder, a bottleneck, and a decoder. A typical convolutional autoencoder is displayed in Fig. 6a. The encoder extracts the features of the input data via a sequence of convolution, nonlinear activation, and pooling (downsampling) operations. The bottleneck is a low dimensional latent representation of the input data, e.g., a $1\,\times\,1\,\times\,512$ vector. The decoder applies another sequence of convolution, nonlinear activation, and upsampling operations to restore the input data from the latent representation.

In Fig. 6a, each blue arrow has a $3\times 3$ zero-padded convolution operation, a ReLU operation, and a batch- normalization operation. Batch normalization is a technique used in neural networks that normalizes the inputs of each layer using the mean and variance of the values in the current batch, thereby improving training speed and stability by reducing the internal covariate shift [55]. The convolution operations extract features at different levels. The ReLU operations generate nonlinear responses. Batch normalization allows neural networks to use higher learning rates and be less sensitive to initialization. The red arrow stands for the max-pooling operation. Max pooling downsamples feature maps by using the maximum value to represent a $2\times 2$ neighborhood. Because of the downsampling operations, the receptive field of $3\times 3$ convolutions is enlarged and large-scale features can be extracted. The green arrow represents an upsampling operation. In particular, a bilinear upsampling with a factor of 2 followed by a $2\times 2$ convolution is used.

**U‑Net** U‑Net [56–58] is the most widely used network for biomedical image processing, including tumor segmentation [59, 60]. It is called “U”-Net because of its U‑shaped architecture design. A typical U‑Net architecture is displayed in Fig. 6b. The left part of the U‑Net is a contraction path, while the right part is an expansion path, both following the typical architectures of CNNs: a sequence of convolution, nonlinear activation, and pooling (i.e., down- or upsampling) operations. U‑Net can be regarded as a modified version of a convolutional autoencoder, where features from the encoder are copied and concatenated to the decoder at different feature levels. The copy operations are represented by the horizontal dashed arrows in Fig. 6b. The concatenation operations stack the features from the encoder and the decoder along the channel dimension (width $\times$ height $\times$ channel for 2D image processing). The copy and concatenation operations allow the U‑Net to employ multilevel features more effectively than the convolutional autoencoder, as fine-scale features lost in the bottleneck can be recovered gradually in the expansion path.

As U‑Nets are very powerful, many different U‑Net variants have been proposed which are characterized by different network topologies, parameters, and modifications that may have included over-optimization to specific datasets without broad generalizability. The nnU-Net or “no new U‑Net” [58] is a particularly important U‑Net-based segmentation pipeline for biomedical segmentation that includes rule-based parameter settings based on dataset characteristics, thus enabling out-of-the-box application to numerous segmentation problems and representing a reference for deep learning-based autosegmentation.

**Others** Many well-known CNNs are currently widely used in various applications. Others include LeNet (the first CNN) [34], AlexNet (the CNN that made deep learning popular) [61], visual geometry group (VGG) networks (commonly used for perceptual loss) [62], GoogLeNet [63], and ResNet (residual learning for better performance) [64].

### Recurrent neural networks (RNNs)

#### General principles of RNNs

While CNNs shine in the domain of spatial data (such as 2D and 3D medical images), recurrent neural networks (RNNs) [65] bring their prowess to sequential data (like text, audio, or tumor respiratory motion information). The MLPs and CNNs introduced above are feed-forward networks, where the information moves in only one direction (from the input layer through multiple hidden layers to the output layer). In contrast, RNNs have network connections that form directed cycles. This structure allows RNNs to maintain a “memory” of previous inputs by incorporating their own output as part of the input for the subsequent step.

**Fig. 7:**
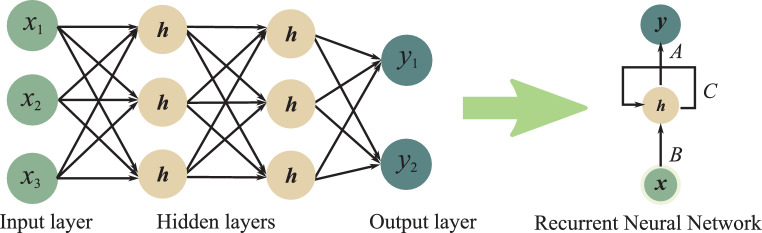
A regular feed-forward MLP network can be converted to an RNN by adding a loop. In the illustration, $\boldsymbol{x}$ is the input layer and $\boldsymbol{y}$ is the output layer. The hidden layers in the feed-forward networks are compressed into one middle layer containing the memory state $\boldsymbol{h}$ in the RNN. A loop is added to $\boldsymbol{h}$ for time-dependent information flow. $A$, $B$, and $C$ are the corresponding network parameters

**Fig. 8:**
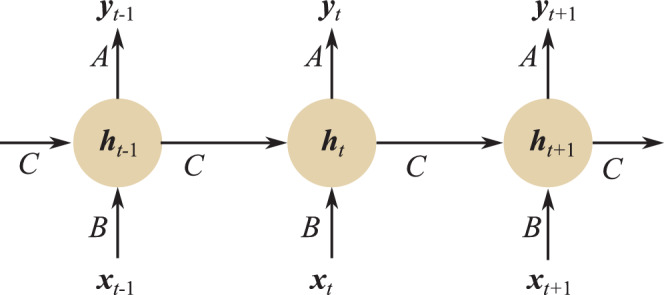
The unrolled RNN architecture. At time $t$, the memory state $\boldsymbol{h}_{t}$ is first updated according to the old internal state $\boldsymbol{h}_{t-1}$ and the current input $\boldsymbol{x}_{t}$ with the parameter $C$, i.e., $\boldsymbol{h}_{t}=f_{C}(\boldsymbol{h}_{t-1},\boldsymbol{x}_{t})$. With $\boldsymbol{h}_{t}$ and $\boldsymbol{x}_{t}$, the current output $\boldsymbol{y}_{t}$ is predicted

As displayed in Fig. 7, a regular feed-forward MLP network can be converted to an RNN by adding a directed loop. In the illustration $\boldsymbol{x}$ is the input layer and $\boldsymbol{y}$ is the output layer. The hidden layers in the feed-forward networks are compressed into one middle layer containing the memory state $\boldsymbol{h}$ in the RNN. A loop is added to $\boldsymbol{h}$ to memorize past time-dependent information. $A$, $B$, and $C$ are corresponding network parameters. The unrolled RNN architecture in Fig. 8 illustrates how an RNN processes sequential data. The key part is that at time $t$, the memory state $\boldsymbol{h}_{t}$ is first updated according to the old internal state $\boldsymbol{h}_{t-1}$ and the current input $\boldsymbol{x}_{t}$ with the parameter $C$, i.e., $\boldsymbol{h}_{t}=f_{C}(\boldsymbol{h}_{t-1},\boldsymbol{x}_{t})$. With this relationship, prior temporal information is captured. With $\boldsymbol{h}_{t}$ and $\boldsymbol{x}_{t}$, the current output $\boldsymbol{y}_{t}$ is predicted with the network parameters $A$.

In the following, a simple example is described for better understanding. Let us consider a simple MLP network with one hidden layer:

7 $$\begin{array}[]{l}\boldsymbol{h}=\tanh(\boldsymbol{W}_{xh}\cdot\boldsymbol{x}),\\ \boldsymbol{y}=\sigma(\boldsymbol{W}_{hy}\cdot\boldsymbol{h}),\end{array}$$

where $\boldsymbol{W}_{xh}$ is the weight matrix between the input layer and the hidden layer, $\boldsymbol{W}_{hy}$ is the weight matrix between the hidden layer and the output layer, $\tanh$ is the specified nonlinear tanh activation function, and $\sigma$ is the nonlinear sigmoid function. An RNN converted from the above MLP network can be represented as

8 $$\begin{array}[]{l}\boldsymbol{h}_{t}=\tanh(\boldsymbol{W}_{hh}\cdot\boldsymbol{h}_{t-1}+\boldsymbol{W}_{xh}\cdot\boldsymbol{x}_{t}),\\ \boldsymbol{y}_{t}=\sigma(\boldsymbol{W}_{hy}\cdot\boldsymbol{h}_{t}),\end{array}$$

where $\boldsymbol{x}_{t}$, $\boldsymbol{y}_{t}$, and $\boldsymbol{h}_{t}$ are time-dependent input, output, and memory state, respectively, with $\boldsymbol{h}_{0}=\boldsymbol{0}$. $\boldsymbol{W}_{hh}$ is the weight matrix between the old memory state $\boldsymbol{h}_{t-1}$ and the current memory state $\boldsymbol{h}_{t}$, which is the added loop connection. Note that the network parameters $\boldsymbol{W}_{hy}$, $\boldsymbol{W}_{xh}$, and $\boldsymbol{W}_{hh}$ do not change over time at the inference phase and they correspond to $A$, $B$, and $C$ in Fig. 7, respectively.

#### Representative networks of RNN

**Long short-term memory (LSTM) networks** To train RNNs, the output errors need to be propagated back to the input through the network layers as well as through time [66]. Backpropagation through time is the regular backpropagation algorithm applied to RNNs with a well-defined order. Because of the repeating application of the chain rule in computing gradients over time, a small gradient will decrease exponentially, leading to the vanishing gradient problem [67]. Therefore, the training is dominated by (the gradients of) early time steps. In addition, think about the challenge of guessing the final word in the following text generation task: “I grew up in Germany…(other long sentences here)… I speak fluent $\_\_\_$.” The latest part of the sentence indicates that the missing word is likely a language. However, to accurately determine which language it is, we must consider the earlier mentioning of Germany. This earlier context is crucial to making a precise prediction, which requires long-term dependency. To solve the above two problems, long short-term memory (LSTM) networks [68, 69] and gated recurrent units (GRUs) [70] were invented.

LSTM [68, 69] introduces three gates to control the information flow, i.e., the forget gate $\boldsymbol{f}_{t}$, the input gate $\boldsymbol{i}_{t}$, and the output gate $\boldsymbol{o}_{t}$. They are mathematically defined as

9 $$\begin{array}[]{l}\boldsymbol{f}_{t}=\sigma(\boldsymbol{W}_{fh}\cdot\boldsymbol{h}_{t-1}+\boldsymbol{W}_{fx}\cdot\boldsymbol{x}_{t}),\\ \boldsymbol{i}_{t}=\sigma(\boldsymbol{W}_{ih}\cdot\boldsymbol{h}_{t-1}+\boldsymbol{W}_{ix}\cdot\boldsymbol{x}_{t}),\\ \boldsymbol{o}_{t}=\sigma(\boldsymbol{W}_{oh}\cdot\boldsymbol{h}_{t-1}+\boldsymbol{W}_{ox}\cdot\boldsymbol{x}_{t}),\end{array}$$

where $\sigma$ is the nonlinear sigma activation function, which maps the internal memory state $\boldsymbol{h}_{t-1}$ and the current input $\boldsymbol{x}_{t}$ into a scalar value between 0 and 1. Hence, these gates can control the information flow between fully off (0) and fully on (1). Compared with the standard RNN definition in Eq. (8), the LSTM definition is characterized as follows:

10 $$\begin{array}[]{l}\tilde{\boldsymbol{c}}_{t}=\tanh(\boldsymbol{W}_{ch}\cdot\boldsymbol{h}_{t-1}+\boldsymbol{W}_{cx}\cdot\boldsymbol{x}_{t}),\\ \boldsymbol{c}_{t}=\boldsymbol{f}_{t}{\otimes}\boldsymbol{c}_{t-1}+\boldsymbol{i}_{t}{\otimes}\tilde{\boldsymbol{c}}_{t},\\ \boldsymbol{h}_{t}=\boldsymbol{o}_{t}{\otimes}\tanh(\boldsymbol{c}_{t}),\\ \end{array}$$

where $\tilde{\boldsymbol{c}}_{t}$ is the candidate cell state, which is a standard RNN like Eq. (8). The previous cell state $\boldsymbol{c}_{t-1}$ and the candidate cell state $\tilde{\boldsymbol{c}}_{t}$ are combined with the forget gate $\boldsymbol{f}_{t}$ and the input gate $\boldsymbol{i}_{t}$ to form the current cell state $\boldsymbol{c}_{t}$, where $\otimes$ is the pointwise multiplication. The new hidden memory state $\boldsymbol{h}_{t}$ is formed by applying the output gate $\boldsymbol{o}_{t}$ to the activated cell state $\boldsymbol{c}_{t}$. With $\boldsymbol{h}_{t}$, the current output is obtained via $\boldsymbol{y}_{t}=\sigma(\boldsymbol{W}_{hy}\cdot\boldsymbol{h}_{t})$.

**Fig. 9:**
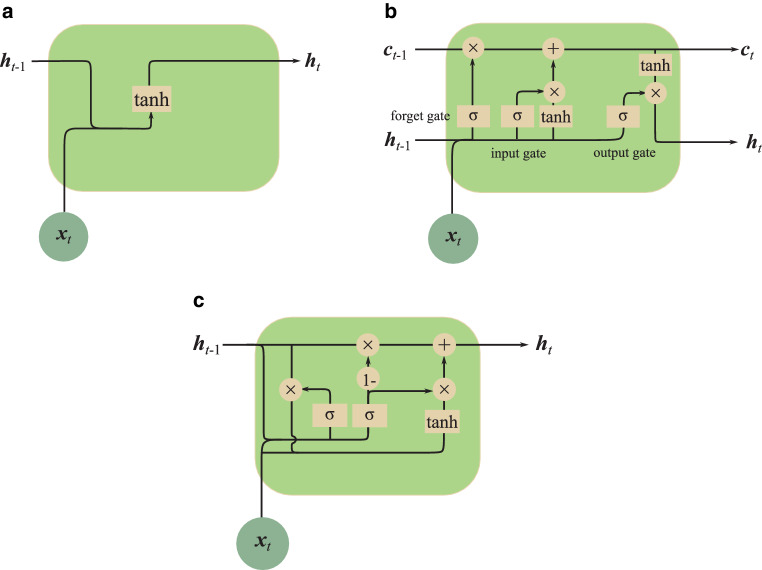
Comparison between a standard RNN unit and an LSTM unit. A simple standard RNN **(a)** uses one tanh activation to combine the previous hidden state $\boldsymbol{h}_{t-1}$ and the current input $\boldsymbol{x}_{t}$ to form the new state $\boldsymbol{h}_{t}$. An LSTM unit **(b)** introduces an additional cell state $\boldsymbol{c}_{t}$ to allow gradients to flow unchanged, which addresses the vanishing gradient problem. In addition, it uses three $\sigma$ activation functions $\sigma(x)=\frac{1}{1+e^{-x}}$ as gates (from left to right in b: forget gate, input gate, and output gate, respectively) and two tanh activation functions $\tanh(x)=\frac{e^{x}-e^{-x}}{e^{x}+e^{-x}}$ to control the information flow, which overcomes the long-term dependency problem. The $\times$ ($\otimes$ in Eq. (10)) and $+$ symbols represent pointwise multiplication and pointwise addition operations, respectively. A gated recurrent unit (GRU; c) is an alternative to LSTM, which uses two $\sigma$ gates and one tanh function. **a** A standard RNN unit, **b** An LSTM unit, **c** A GRU unit

LSTM networks address the vanishing gradient problem through their unique cell state ($\boldsymbol{c}_{t}$) design, which allows gradients to flow unchanged, thus ensuring stable training over many time steps. Additionally, they overcome long-term dependency issues with the three gates that regulate information flow, enabling the network to retain or forget information selectively, thereby efficiently capturing long-term relationships in data. (Note that GRU [70] is a simpler alternative to LSTM using two gates only, as displayed in Fig. 9c.) LSTM networks have been successfully applied to various radiation oncology tasks [71], e.g., for predicting respiratory signal using RNNs for thoracoabdominal tumor radiotherapy [72] and for predicting glioma growth [73, 74].

### Transformers

#### General principles of transformers

Transformers have redefined the boundaries of what can be achieved by deep learning. Transformers can weigh the importance of different parts of the input data, regardless of the distance between them, thus rendering them beneficial in natural language processing (NLP) and other sequence-related tasks. Central to transformers is the mechanism of tokenization and the innovative use of self-attention mechanisms characterized by the variables query ($\boldsymbol{Q}$), key ($\boldsymbol{K}$), and value ($\boldsymbol{V}$) [26].

Tokenization is the first step in processing data using a transformer model. In this step, input data, typically text, is converted into tokens. Each token is a piece of the original data, like a word or part of a word, that can be individually processed. These tokens are then converted into numerical vectors using embedding techniques like Word2vec [75, 76]. Because text tokens can be embedded into numerical vectors, a complete input text can be represented by a matrix $\boldsymbol{X}$, which is a stack of such token vectors. If the text has $n$ tokens and each token is represented by a $d$-dimensional vector, $\boldsymbol{X}$ would be an $n\times d$ matrix.

The core innovation of the transformer architecture is the self-attention mechanism, which allows the model to weigh the importance of different tokens in a sequence relative to each other. The mechanism computes this attention using three main variables: query ($\boldsymbol{Q}$), key ($\boldsymbol{K}$), and value ($\boldsymbol{V}$). These are derived from the input tokens and play crucial roles in shaping the output of the attention process. Query ($\boldsymbol{Q}$) is a set of vectors that is used to probe the sequence. It represents the current token in context and looks for relevant information across the sequence. Key ($\boldsymbol{K}$) is a set of vectors that corresponds to each token in the sequence, against which the queries are compared. The relationship between a query and all keys determines the weighting of significance for each token in the sequence. Once the model determines the tokens to focus on (as guided by the strength of the query–key relationships), the value vectors ($\boldsymbol{V}$) of those tokens are aggregated to form the output of the attention mechanism.

To create the $\boldsymbol{Q}$, $\boldsymbol{K}$, and $\boldsymbol{V}$ matrices, three sets of weight matrices need to be initialized: $\boldsymbol{W}^{\boldsymbol{Q}}$, $\boldsymbol{W}^{\boldsymbol{K}}$, and $\boldsymbol{W}^{\boldsymbol{V}}$. These are trainable parameters of the model and are usually learned during the training process. The dimensions of these matrices depend on the desired dimensionality of the $\boldsymbol{Q}$, $\boldsymbol{K}$, and $\boldsymbol{V}$ vectors (denoted as $d_{k}$ for $\boldsymbol{Q}$ and $\boldsymbol{K}$ and as $d_{v}$ for V). Typically, $\boldsymbol{W}^{\boldsymbol{Q}}$, $\boldsymbol{W}^{\boldsymbol{K}}$, and $\boldsymbol{W}^{\boldsymbol{V}}$ are dimensions of $d\times d_{k}$, $d\times d_{k}$, and $d\times d_{v}$, respectively. With the input matrix $\boldsymbol{X}$ and the trainable weight matrices, the $\boldsymbol{Q}$, $\boldsymbol{K}$, and $\boldsymbol{V}$ matrices are computed by matrix multiplication, i.e., $\boldsymbol{Q}=\boldsymbol{X}\boldsymbol{W}^{\boldsymbol{Q}}$, $\boldsymbol{K}=\boldsymbol{X}\boldsymbol{W}^{\boldsymbol{K}}$, and $\boldsymbol{V}=\boldsymbol{X}\boldsymbol{W}^{\boldsymbol{V}}$.

Given the computed $\boldsymbol{Q}$, $\boldsymbol{K}$, and $\boldsymbol{V}$ matrices, the attention scores between all pairs of queries and keys are calculated, typically using a scaled dot-product attention module [26] defined as the following:

11 $$\textrm{attention}(\boldsymbol{Q},\boldsymbol{K},\boldsymbol{V})= \textrm{softmax}\left(\frac{\boldsymbol{Q}\cdot\boldsymbol{K}^{\top}}{\sqrt{d}}\right)\boldsymbol{V},$$

where a division of $\sqrt{d}$ is used for rescaling to get stable gradient computation during training. The softmax function is defined as

12 $$\delta(\boldsymbol{x})=\frac{e^{x_{i}}}{\sum_{j=0}^{K-1}e^{z_{j}}} \textrm{ for }i=0,{\ldots},K-1, \textrm{ and }\boldsymbol{x}=(x_{1},x_{2},{\ldots},x_{k-1}).$$

**Fig. 10:**
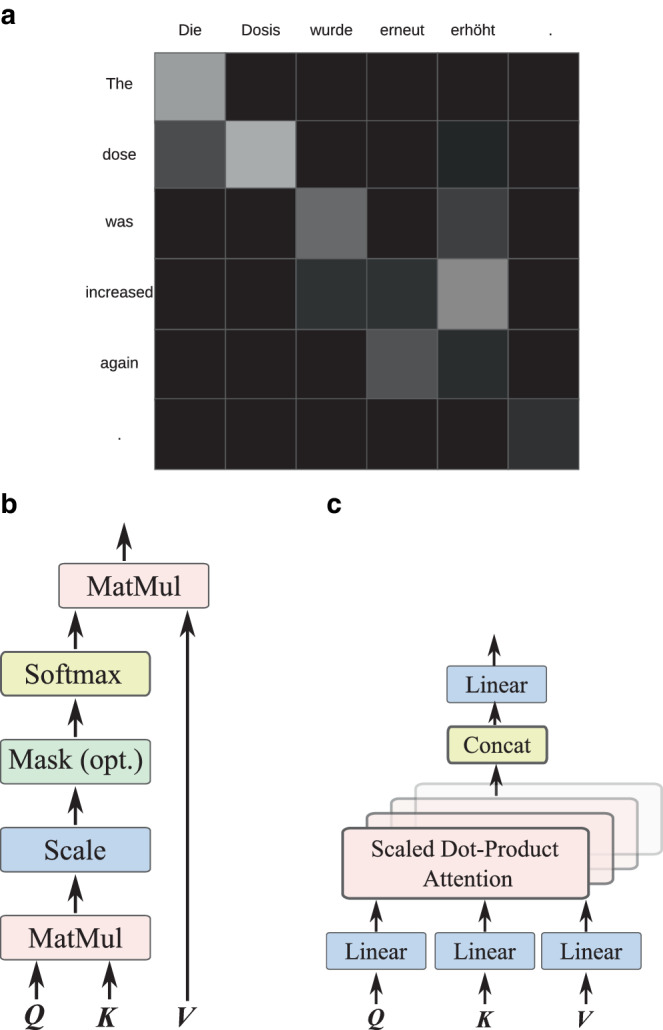
Attention mechanism in transformer models: **a** the attention weights in a German-to-English translation example [77]; **b** the structure of a scaled dot-product attention layer, where “MatMul” stands for matrix multiplication and “Mask (opt.)” stands for optional mask operation; **c** the structure of a multihead attention module. **a** Attention example, **b** Scaled dot-product attention, **c** Multihead attention

The softmax function normalizes all the vector elements to the range [0, 1] and the sum of normalized elements to 1, like probabilities, which is beneficial to keep the scale of the attention output. This scaled dot-product attention module is illustrated in Fig. 10b.

In Fig. 10b, an optional mask operation is added. This is necessary for some tasks like text generation, where the output sentence of an attention module is related to the previously generated words but not to words generated in the future. Therefore, a mask is necessary to avoid attention to unavailable context.

In order to fully employ the features in the sentence from different aspects (e.g., different semantic connections or syntactic structure), multiple copies of the scaled dot-product attention modules are assembled together to form a multihead attention, which is illustrated in Fig. 10c. Such multihead attention blocks become essential components of modern transformer networks. Because of the attention mechanism, transformer networks are capable of global context awareness. This global context awareness, together with introducing auxiliary techniques as well as increasing the quantity and quality training data, has boosted the development of NLP models, leading to the prosperity of language models including Bidirectional Encoder Representations from Transformers (BERT) [78] and Generative Pre-Trained Transformer‑4 (GPT-4), which are promising for decision-making support in radiation oncology [79–81].

The potential of the transformer architecture extends beyond NLP to computer vision tasks and medical image analysis [82], thanks to the development of the vision transformer (ViT) architecture [83]. Transformers have been applied to enhance the analysis of medical images, such as MRI and CT scans [84–87]. These applications capitalize on the global context awareness of transformers to reveal subtle patterns indicative of pathological conditions or treatment response. Noteworthy applications in radiation oncology [86, 87] include the TransUNet work [86] in which transformers were employed to improve the accuracy of tumor segmentation in CT scans, and the Multi-transSP work in which transformers are used to extract multimodal information for survival prediction. Both applications showcase their potential in aiding radiation oncologists in treatment planning and assessment.

Compared to RNNs, transformers have advantages in parallelization and long-distance dependencies: (i) unlike RNNs, which process data sequentially, transformers process entire sequences simultaneously. This allows for significantly more efficient training because the computations can be parallelized, making transformers particularly well suited for modern computing hardware like GPUs. (ii) Transformers use self-attention mechanisms to weigh the importance of each part of the input data relative to each other part, regardless of their positions in the sequence. This allows them to capture long-range dependencies more effectively than RNNs, which can struggle with such dependencies due to vanishing and exploding gradient issues. Because of these factors, transformers have largely overshadowed RNNs in many advanced applications. Nevertheless, RNNs are not obsolete. Since transformers typically require a large amount of training data and are computationally expensive, RNNs with a simpler architecture continue to have their importance in resource-constrained environments. Moreover, transformers process sequential data in a pseudotemporal manner, whereas the inherent sequential nature of RNNs is more appropriate for tasks where the sequence order strictly matters. Therefore, some hybrid networks such as Transformer-XL [88] and fast autoregressive transformers [89] that integrate RNNs and transformers together have been proposed.

#### Representative networks of transformers

**Fig. 11:**
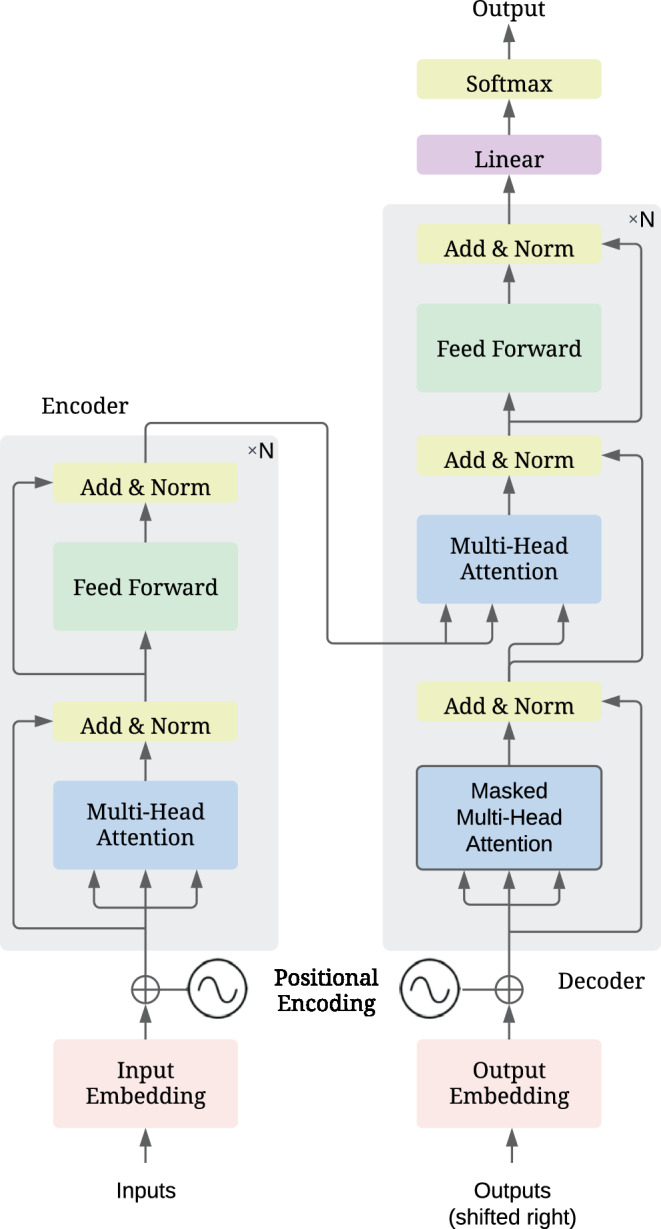
The typical transformer architecture [26], which consists of an encoder (*left*) and decoder (*right*). The encoder and decoder each consist of $N$ (e.g., $N=6$) copies of the same modules, where each module shares the same architecture but with different weights

**Original transformer model** The typical transformer architecture, introduced by Vaswani et al. for NLP tasks like language translation [26], is structured as an encoder–decoder framework, as illustrated in Fig. 11.

The encoder has one input (e.g., the source language text in translation tasks) and one output to the decoder (keys and values for the decoder). The input layer consists of two parts: text embedding and positional encoding. The input text is first embedded into a vector representation using embedding techniques like Word2vec [75, 76], so that it can be processed by networks. Unlike RNNs, which process words one after another in a sequential manner, transformers can process multiple words *simultaneously in parallel*, which requires positional encoding to tell the position of each word. Like the embedding of words, a vector (constructed by sine and cosine values of different frequencies) [26] is used to represent the position of each word and this positional vector is directly added to the word vector as the input of the encoder. The main body of the encoder consists of $N$ (e.g., $N=6$) copies of the same modules, where each module shares the same architecture but with different weights.

The decoder has two inputs: one from the encoder and the other being the previous output of the decoder (e.g., the already generated target language text in translation tasks, which corresponds to an autoregressive property). Enabled by its self-attention, the encoder undertakes the processing of the input sequence, thereby producing a contextualized representation. This functionality allows the encoder to model interactions among different segments of the input sequence. The decoder then utilizes this representation to generate output predictions. The attention mechanisms (self-attention present in both the encoder and decoder as well as cross-attention connecting each encoder and decoder) play a crucial role in the model efficiently capturing long-range dependencies and contextual information, addressing issues like the vanishing gradient problem associated with traditional RNNs.

**Fig. 12:**
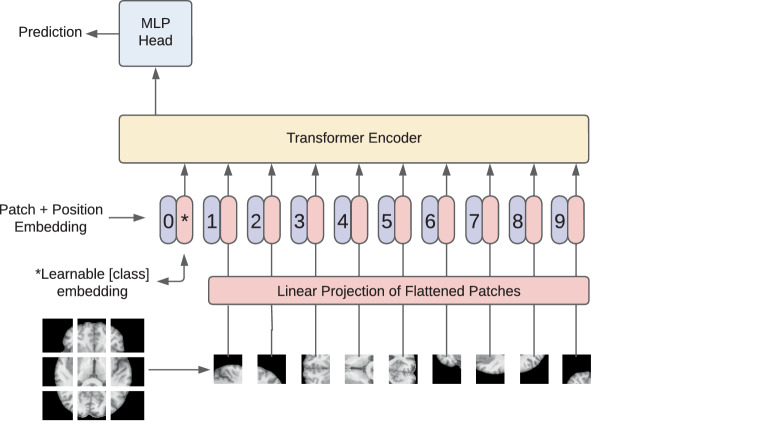
Illustration of the vision transformer (*ViT*) architecture. ViT transforms input images into a sequence of flattened patches which are then processed through a transformer architecture. Each patch undergoes self-attention mechanisms, allowing the model to capture global dependencies and contextual information. The learned representations are subsequently used for various downstream vision tasks, showcasing the efficacy of the transformer paradigm in image understanding and feature extraction

**Vision transformer (ViT)** ViT [83], as illustrated in Fig. 12, presents a broadly adopted modification of the transformer architecture in computer vision tasks. In this model, images are processed as sequences of patches (one complete image is cut into small patches and stored in an order), which are then linearly embedded to be passed through transformer encoder layers. The integration of the transformer approach into computer vision highlights its ranging utility. Based on ViT, many other transformer networks are being developed for medical imaging processing, including TransUNet [86], Swin-UNet [90], and Swin-UNETR [91].

### Generative adversarial networks (GANs)

#### General principles of GANs

Generative adversarial networks (GANs) have achieved huge success since they were introduced by J. Goodfellow et al. [92]. GANs are a special type of neural network consisting of two neural networks trained simultaneously: one generator $G$ and one discriminator $D$. The generator and the discriminator engage in a two-player min–max game. The generator tries to produce data that look as real as possible, while the discriminator tries to get better at distinguishing real data from fake data. The process continues iteratively until equilibrium is reached, where the generator produces data almost identical to real data and the discriminator cannot differentiate between them.

**Fig. 13:**
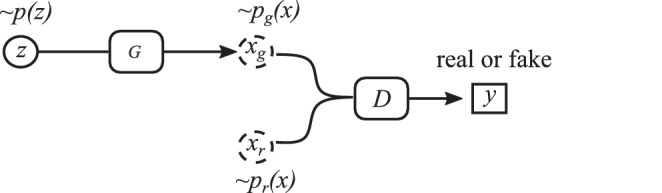
Architecture of the vanilla GAN in [92]: $z$ is the input of the generator $G$, which is a random signal such as Gaussian noise; $x_{r}$ is a sample from real images; the generator $G$ concerts the random signal into a synthetic image $x_{g}$, which is as realistic as $x_{r}$. The discriminator $D$ is optimized to tell the synthetic images from real images

The vanilla GAN’s architecture [92] is displayed in Fig. 13. The input of the generator, $z$, is a random signal with a prior distribution $p(z)$, e.g., a Gaussian or uniform distribution. The output of the generator, $x_{g}$, which has a distribution of $p_{g}(x)$, should be close to a real sample $x_{r}$ from a distribution $p_{r}(x)$. The objective of the discriminator is to tell whether an input signal is a real sample or a generated fake one, while the generator is trained to confuse the discriminator as much as possible.

#### Representative networks of the GAN type

**Fig. 14:**
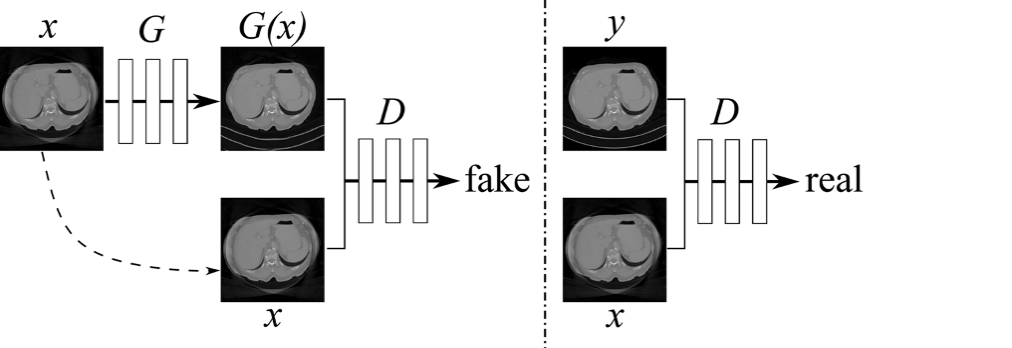
Architecture of Pix2pixGAN [93], a conditional GAN, in the application of CT image processing [50]. The generator $G$, which is typically a U-Net, converts a corrupted CT image into an artifact-free image. The discriminator $D$ learns to distinguish the output image from the target image conditioned on the input image

In addition to image generation from noise (Fig. 13), GANs are capable of image-to-image translation, i.e., producing an image given another image (conditional GANs) [93–95]. The most widely known GANs for image-to-image translation are Pix2pixGAN [93] and CycleGAN [94]. Pix2pixGAN requires paired images for image translation, whereas CycleGAN allows unpaired images. In Fig. 14, the architecture of Pix2pixGAN in the application of CT image processing [50] is illustrated: the generator $G$, which is typically a U-Net [56], converts a corrupted CT image into an artifact-free image. The discriminator $D$ learns to distinguish the output image from the target image conditioned on the given input image.

Due to the great power of GANs in image generation, they have been widely used in various medical applications [95], including the field of radiation oncology. For example, GANs are commonly applied for synthetic CT generation from CBCT [96–98] or MRI images [99, 100]. GANs can also be applied for image segmentation and registration in prostate cancer radiotherapy [101].

### Diffusion-based generative models

#### General principles of diffusion-based generative models

**Fig. 15:**
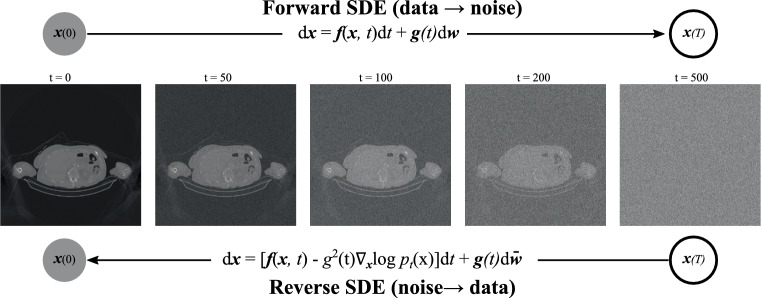
General pipeline of diffusion-based generative models [102]. In the forward diffusion process, noise is added to corrupt the data slowly into random noise. In the reverse diffusion process, a random noise sample is picked and gradually denoised into a clean image for image generation. A network is trained to estimate the score function, i.e., the gradient of data distribution with respect to space $\nabla_{x}\log p_{t}(\boldsymbol{x})$, to guide the reverse process for image generation from a random noise sample

Diverging from traditional generative models, diffusion-based generative approaches [102–106] model data generation as a stochastic process, akin to particles undergoing random diffusion in a fluid. By reversing this process, such models can generate new data instances by starting from a noise sample and gradually refining it through iterative steps. A general pipeline of diffusion-based generative models is displayed in Fig. 15, where stochastic noise is added to an image in the forward diffusion process multiple times (500 times in the example) to corrupt the data slowly into random noise. For image generation, a random noise sample is picked and a reversed diffusion process is performed to gradually denoise a noisy image into a clean image.

The forward diffusion process follows a stochastic differential equation (SDE) in the following form [102]:

13 $$\textrm{d}\boldsymbol{x}=\boldsymbol{f}(\boldsymbol{x},t) \textrm{d}t+\boldsymbol{g}(t) \textrm{d}\boldsymbol{w},$$

where $\boldsymbol{f}(\boldsymbol{x},t)$ is the drift coefficient, $\boldsymbol{g}(t)$ is the diffusion coefficient, and $\boldsymbol{w}$ represents the standard Brownian motion (i.e., a random vector in magnitude and direction). While $\boldsymbol{f}(\boldsymbol{x},t) \textrm{d}t$ describes the deterministic change of an image over time, $\boldsymbol{g}(t) \textrm{d}\boldsymbol{w}$ describes the stochastic change of an image over time.

As the reverse of a diffusion process is also a diffusion process, the reverse-time SDE process has the following form [102, 107]:

14 $$\textrm{d}\boldsymbol{x}=\left[\boldsymbol{f}(\boldsymbol{x},t)-\boldsymbol{g}(t)^{2}\nabla_{\boldsymbol{x}}\log p_{t}(\boldsymbol{x})\right] \textrm{d}t+\boldsymbol{g}(t) \textrm{d}\bar{\boldsymbol{w}},$$

where $\bar{\boldsymbol{w}}$ is the standard Brownian motion when time flows backwards, which is implemented in the same way as the forward Brownian motion, as a random process; $p_{t}(\boldsymbol{x})$ is the data distribution probability at time $t$ and $\nabla_{x}\log p_{t}(\boldsymbol{x})$ is the gradient of the data distribution over the space; and $\nabla_{\boldsymbol{x}}\log p_{t}(\boldsymbol{x})$ is called the score function in score-based generative models [103, 108]. It tells a model in which direction to go to increase the probability of data. Conventionally, the data distribution probability needs to be normalized, i.e., $p^{\prime}_{t}(\boldsymbol{x})=\frac{p_{t}(\boldsymbol{x})}{s}$, to let all probabilities sum up to 1, where $s$ is the integral/sum of $p_{t}(\boldsymbol{x})$. With the logarithm operation and gradient operation in the score function $\nabla_{\boldsymbol{x}}\log p_{t}(\boldsymbol{x})$, score-based models do not have to be normalized because $\nabla_{\boldsymbol{x}}\log p_{t}(\boldsymbol{x})=\nabla_{\boldsymbol{x}}\log p^{\prime}_{t}(\boldsymbol{x})-C$, where $C$ is a constant value which does not change the optimization problem. Therefore, the formulation of the score function provides huge flexibility in choosing model architectures.

The above reverse-time SDE shares the same marginals as the following ordinary differential equation (ODE), namely the probability flow ODE [102]:

15 $$\textrm{d}\boldsymbol{x}=\left[\boldsymbol{f}(\boldsymbol{x},t)-\frac{1}{2}\boldsymbol{g}(t)^{2}\nabla_{\boldsymbol{x}}\log p_{t}(\boldsymbol{x})\right] \textrm{d}t,$$

where the stochastic Brownian motion is removed and, hence, the ODE is deterministic.

In diffusion-based generative models, a network can learn the score function $\nabla_{x}\log p_{t}(\boldsymbol{x})$ to guide the reverse diffusion process. Typically, a noise-conditional or time-conditional U‑Net with attention mechanisms [103] is applied to learn the score function. With the learned score function, the reverse-time SDE and the probability flow ODE can both be solved by various numerical methods, such as numerical SDE solvers [102, 109], numerical ODE solvers [102, 105, 110, 111], annealed Langevin dynamics [103], and predictor–corrector methods [102].

The applications of diffusion models in radiation oncology are being explored [112]. They can be applied to multicontrast MRI image translation and MRI–CT translation [113], fast MRI reconstruction [114, 115], brain MRI image synthesis for training brain tumor segmentation models [116], and diffusion MRI denoising [117].

#### Representative networks of diffusion-based generative models

**Denoising diffusion-based probabilistic models (DDPMs)** DDPMs [104, 118] represent the first well-known diffusion-based generative models. DDPMs are a special discrete form of SDE diffusion models (Eq. (13)) with the following specific forward SDE [102]:

16 $$\textrm{d}\boldsymbol{x}=-\frac{1}{2}\beta(t)\boldsymbol{x} \textrm{d}t+\sqrt{\beta(t)} \textrm{d}\boldsymbol{w}.$$

and the corresponding reverse SDE form

17 $$\textrm{d}\boldsymbol{x}=-\frac{1}{2}\beta(t)(\boldsymbol{x}_{t} \textrm{d}t-\nabla_{x_{t}}\log p_{t}(\boldsymbol{x}_{t})] \textrm{d}t+\sqrt{\beta(t)} \textrm{d}\boldsymbol{w}.$$

DDPMs [104, 118] are constructed by two Markov chains: a forward chain that perturbs data to noise and a reverse chain that converts noise back to data. A Markov process assumes that the next state of a process only depends on the present state and not on the past states. Given an initial clean image $\boldsymbol{x}_{0}=\boldsymbol{x}$, typically, Gaussian perturbation is applied in the forward Markov chain with the following transition kernel:

18 $$q(\boldsymbol{x}_{t}|\boldsymbol{x}_{t-1})=\mathcal{N}\left(\boldsymbol{x}_{t};\sqrt{1-\beta_{t}}\boldsymbol{x}_{t-1},\beta_{t}\boldsymbol{I}\right),$$

where $\mathcal{N}$ denotes the Gaussian distribution, $\boldsymbol{I}$ is an identity vector, and $\beta_{t}\in(0,1)$ is a preset hyperparameter for training. It means that at each pixel index $(i,j)$ of the 2D image $\boldsymbol{x}_{t}$, a random Gaussian noise with the mean value of $\sqrt{1-\beta_{t}}\boldsymbol{x}_{t-1}(i,j)$ and the variance of $\beta_{t}$ is added. Denoting $\alpha_{t}:=1-\beta_{t}$ and $\bar{\alpha}_{t}:=\Pi_{s=0}^{t}\alpha_{s}$, Eq. (18) leads to the following:

19 $$q(\boldsymbol{x}_{t}|\boldsymbol{x}_{0})=\mathcal{N}\left(\boldsymbol{x}_{t};\sqrt{\bar{\alpha}_{t}}\boldsymbol{x}_{0},(1-\bar{\alpha}_{t})\boldsymbol{I}\right).$$

Therefore, the relationship between $\boldsymbol{x}_{t}$ and $\boldsymbol{x}_{0}$ is

20 $$\boldsymbol{x}_{t}=\sqrt{\bar{\alpha}_{t}}\boldsymbol{x}_{0}+\sqrt{1-\bar{\alpha}_{t}}\boldsymbol{\epsilon},$$

where $\boldsymbol{\epsilon}$ is a standard Gaussian noise $\boldsymbol{\epsilon}\thicksim\mathcal{N}(0,\boldsymbol{I})$. When $T$ is large enough ($T$ is the total number of time steps in the forward process), we have $\bar{\alpha}_{T}\approx 0$ and $\boldsymbol{x}_{T}$ becomes pure Gaussian noise (which is the latent representation of the image).

For the reverse process, which is image generation, we need to learn the reverse Markov chain in the following form:

21 $$p_{\boldsymbol{\theta}}(\boldsymbol{x}_{t-1}|\boldsymbol{x}_{t})=\mathcal{N}\left(\boldsymbol{x}_{t-1};\boldsymbol{\mu}_{\boldsymbol{\theta}}(\boldsymbol{x}_{t},t),\boldsymbol{\Sigma}_{\boldsymbol{\theta}}(\boldsymbol{x}_{t},t)\right),$$

where $\boldsymbol{\theta}$ is the model parameter set and $\boldsymbol{\mu}_{\boldsymbol{\theta}}(\boldsymbol{x}_{t},t)$ and $\boldsymbol{\Sigma}_{\boldsymbol{\theta}}(\boldsymbol{x}_{t},t))$ are mean and variance vectors parameterized by deep neural networks, respectively. With such a learnable reverse transition kernel, an image can be generated from Gaussian noise.

**Denoising diffusion implicit models (DDIMs)** DDIMs [105] extend the original DDPMs to non-Markovian processes with the following forward process:

22 $$q(\boldsymbol{x}_{1},{\ldots},\boldsymbol{x}_{T}|\boldsymbol{x}_{0})=\prod_{t=1}^{T}q(\boldsymbol{x}_{t}|\boldsymbol{x}_{t-1},\boldsymbol{x}_{0}),$$

where $\boldsymbol{x}_{t}$ depends on not only $\boldsymbol{x}_{t-1}$ but also $\boldsymbol{x}_{0}$. Therefore, this is a non-Markovian process. The reverse process is tractable when conditioned on the initial image $\boldsymbol{x}_{0}$:

23 $$\begin{array}[]{l}q_{\sigma}(\boldsymbol{x}_{t-1}|\boldsymbol{x}_{t},\boldsymbol{x}_{0})=\mathcal{N}\left(\boldsymbol{x}_{t-1};\tilde{\boldsymbol{\mu}}_{t}(\boldsymbol{x}_{t},\boldsymbol{x}_{0}),\sigma_{t}^{2}\boldsymbol{I}\right),\\ \tilde{\boldsymbol{\mu}}_{t}(\boldsymbol{x}_{t},\boldsymbol{x}_{0})=\sqrt{\bar{\alpha}_{t-1}}\boldsymbol{x}_{0}+\sqrt{1-\bar{\alpha}_{t-1}-\sigma_{t}^{2}}\cdot\frac{\boldsymbol{x}_{t}-\sqrt{\bar{\alpha}_{t}}\boldsymbol{x}_{0}}{\sqrt{1-\bar{\alpha}_{t}}}.\end{array}$$

The above formulation covers DDPMs and DDIMs as special cases, where DDPMs correspond to setting $\sigma_{t}^{2}=\frac{1-\bar{\alpha}_{t-1}}{1-\bar{\alpha}_{t}}\beta_{t}$, whereas DDIMs correspond to setting $\sigma_{t}^{2}=0$. Since $\sigma_{t}^{2}=0$, no stochastic noise is added and the reverse process is fully deterministic for DDIMs. In other words, a model can be trained with an arbitrary number of forward steps but only some intermediate steps are necessary in the reverse process for image generation. Therefore, DDIMs can accelerate image generation with similar image quality.

**Conditional latent diffusion models** Conditional latent diffusion models (LDMs) [119, 120] harness the power of a diffusion process within a latent space rather than directly in the high-dimensional pixel space to generate images conditionally based on various inputs like text or structured labels. In the latent space, the diffusion process can be mathematically modeled by a Markov chain of latent variables $\{\boldsymbol{z}_{t}\}_{t=0}^{T}$, where $\boldsymbol{z}_{0}$ is derived from the data distribution and $\boldsymbol{z}_{T}$ is typically noise from a known distribution such as a Gaussian distribution. The transition from $\boldsymbol{z}_{t-1}$ to $\boldsymbol{z}_{t}$ is governed by a Gaussian transition probability, formally expressed as

24 $$p(\boldsymbol{z}_{t}|\boldsymbol{z}_{t-1})=\mathcal{N}\left(\boldsymbol{z}_{t};\sqrt{1-\beta_{t}}\boldsymbol{z}_{t-1},\beta_{t}\boldsymbol{I}\right),$$

where $\beta_{t}$ is a variance schedule that guides the addition of noise over the diffusion step $t$ and $\boldsymbol{I}$ represents the identity matrix. This step-by-step noising process allows for an effective representation and manipulation of the data in its latent form, significantly optimizing computational efficiency.

To reverse this process for image generation, conditional LDMs employ a neural network parameterized as $\boldsymbol{\theta}$ to model the reverse diffusion from noise to data. This is described by the conditional probability $p_{\boldsymbol{\theta}}(\boldsymbol{z}_{t-1}|\boldsymbol{z}_{t},\boldsymbol{c})$, which is approximated by the network learning to denoise the data:

25 $$p_{\boldsymbol{\theta}}(\boldsymbol{z}_{t-1}|\boldsymbol{z}_{t},\boldsymbol{c})=\mathcal{N}\left(\boldsymbol{z}_{t-1};\boldsymbol{\mu}_{\boldsymbol{\theta}}(\boldsymbol{z}_{t},t,\boldsymbol{c}),\boldsymbol{\Sigma}_{\boldsymbol{\theta}}(\boldsymbol{z}_{t},t,\boldsymbol{c})\right).$$

In the above equation, $\boldsymbol{\mu}_{\boldsymbol{\theta}}$ and $\boldsymbol{\Sigma}_{\boldsymbol{\theta}}$ are functions learned during training and $\boldsymbol{c}$ represents the conditioning variables such as text descriptions or other labels. Compared with Eq. (21), Eq. (25) works in the latent space $\boldsymbol{z}$ instead of in the original data space $\boldsymbol{x}$, with the additional condition $\boldsymbol{z}$. This mathematical framework allows LDMs to perform tasks such as text-to-image synthesis, class-conditional image generation, and even complex video synthesis with remarkable fidelity [120].

### Deep reinforcement learning

#### General principles of deep reinforcement learning

Reinforcement learning [121–123] is a general framework within which agents learn to perform actions in an environment to maximize a reward. Unlike supervised learning, reinforcement learning needs to learn with highly delayed supervised information (e.g., success or failure of the decision is available only after multiple time steps) and has to deal with sequential decisions [123]. The two main components of reinforcement learning are the environment, which represents the problem to be solved, and the agent, which represents the AI model. The agent has a policy $\pi_{\boldsymbol{\theta}}(s,a)$ to determine which action $a$ to take based on the current state $s$. This action will interact with the environment, and the environment will provide feedback (reward) to the agent to adjust the agent’s behaviors based on the defined immediate reward function $r$. The goal of reinforcement learning is to improve the policy $\pi_{\boldsymbol{\theta}}(s,a)$ to maximize the accumulated rewards. Reinforcement learning can be separated into two general categories [122]: model-free and model-based algorithms. Model-free reinforcement learning algorithms do not create a parameterized model of the environment’s transition function to make predictions of future states and rewards. In real applications, such a parameterized model is typically not available. Therefore, model-free reinforcement learning algorithms are generally more useful in practice than model-based ones. Model-free algorithms can further be categorized into value-based [124, 125] and policy-based [126] methods, which determine the optimal reward value and policy, respectively.

**Fig. 16:**
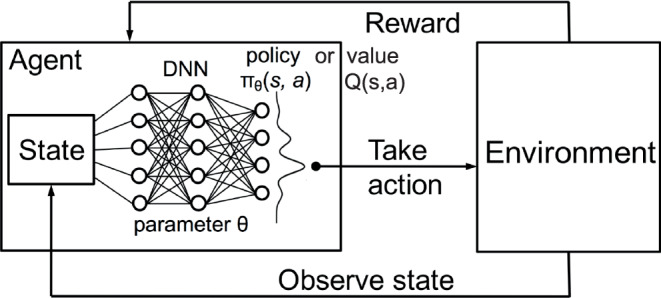
Architecture of deep reinforcement learning. Reinforcement learning in general consists of two main components: agent and environment. The agent has a certain polity $\pi$ to take an action $a$ based on the current observed state $s$. The agent will interact with the environment. With the action, the environment provides certain feedback (reward or penalty) to the agent. The agent aims to obtain the maximum reward from the environment. Deep reinforcement learning uses a deep neural network to estimate the optimal reward (e.g., the Q function $Q(s,a)$) or the optimal policy $\pi_{\boldsymbol{\theta}}(s,a)$ with an input state $s$, which guides the agent to obtain the maximum cumulative reward after a series of actions

For deep reinforcement learning, an agent is typically a deep neural network (DNN) with a parameter set $\boldsymbol{\theta}$, which is used to estimate the optimal reward value or policy given the current state $s$, as displayed in Fig. 16. A policy network directly maps the states of the environment to actions. The network’s output is a policy, which dictates the probability distribution of selecting each possible action from a given state, e.g., the policy networks in Alpha Go [127]. This is known as a stochastic policy, which can be beneficial in environments in which exploration is important. Policy networks can also output deterministic actions [128], where the policy directly specifies the action to be taken without any probabilistic behavior. A value network, on the other hand, typically estimates the reward value of taking a particular action in a given state [125]. The estimated function is called the state-value function or, as more commonly known, the Q‑function (denoted as $Q(s,a)$) [124], and such learning is well known as deep Q‑learning, which has been demonstrated to successfully achieve human-level control in playing Atari 2600 games directly from pixel data [125]. The optimal Q‑function value at a given state $s$ and a given action $a$ is further denoted as $Q^{\ast}(s,a)$, which is mathematically defined as

26 $$Q^{\ast}(s,a)=\max_{\pi}\mathbb{E}[\sum_{t\geq 0}\gamma_{t}r_{t}|s_{0}=s,a_{0}=a,\pi],$$

where $r_{t}$ is the immediate reward at time $t$ and $\gamma_{t+1}$ is a discount factor for the maximum future reward obtained by the next state $s_{t+1}$ and action $a_{t+1}$. The optimal $Q^{\ast}$ functions have the following recurrence relation, i.e., the Bellman optimality equation [129]:

27 $$Q^{\ast}(s,a)=\mathbb{E}[r_{t}+\gamma_{t+1}\max_{a_{t+1}}Q^{\ast}(s_{t+1},a_{t+1})].$$

When we use a DNN to estimate the optimal $Q^{\ast}$ function in deep Q‑learning, Eq. (27) allows us to start with a random $Q$ function (i.e., random weights $\boldsymbol{\theta}$ for the DNN) and update the network iteratively with the Bellman optimality equation as the loss. With sufficient iterations, $Q$ converges to $Q^{\ast}$ [130].

Deep reinforcement learning has been investigated in many medical imaging tasks such as landmark detection [131–133], image registration [134, 135], and view selection [136]. In the field of radiation oncology, deep reinforcement learning also has great potential in tasks like lesion detection [137], cancer classification [138], smart patient scheduling [139], automated radiation adaptation [140, 141], and general decision support in oncology [142].

#### Representative networks of deep reinforcement learning

**Deep Q‑networks (DQNs)** Deep Q‑networks (DQNs) are nonlinear approximators in deep Q‑learning for the optimal Q functions: $Q(s,a;\boldsymbol{\theta})\approx Q(s,a)$, where $\boldsymbol{\theta}$ is the network parameter set. All the networks introduced in the above subsections (3.1 to 3.6) can be plugged as DQNs for deep Q‑learning. DQNs incorporate several key design elements to enhance learning stability and efficiency: *experience replay* and *target networks* [125]. Experience replay involves storing agent experiences at each time step in a replay buffer and randomly sampling mini-batches from this buffer to update the network. This method breaks the correlation between consecutive learning samples, thus stabilizing training. Target networks, on the other hand, are clones of the main network that are held fixed for several updates, providing consistent targets during temporal difference learning. This separation reduces the volatility of updates, thereby further stabilizing the learning process. Together, these mechanisms enable DQNs to learn effective policies in complex and dynamic environments.

An exemplary application of DQNs is anatomical landmark detection [131], which is beneficial for organ at risk (OAR) autocontouring in radiation therapy. Here, a DQN (which is a CNN) is used to locate the target organ region, i.e., the cuboid image subvolume to be subsequently processed by the organ-specific autosegmentation model. In this application, the state $s$ represents the location of the center of the current volume of interest (VOI), the action $s$ is to move the VOI to its neighboring position (moving up, down, left, right, front, and back), and $r$ is a scalar reward function which calculates the distance between the current VOI location and the target organ center. The trained model has a policy $\pi$ to tell the VOI which direction to move in at a given location, which forms a searching trajectory from the start location to the target organ.

**Actor–critic methods** Actor–critic methods [143, 144] represent a powerful class of algorithms in deep reinforcement learning that synergistically combine the strengths of policy- and value-based approaches. These methods utilize two main components: the *actor*, which is responsible for learning the policy function, and the *critic*, which evaluates the policy by learning the value function. The actor updates the policy distribution in the direction suggested by the critic, aiming to maximize expected rewards, whereas the critic assesses the actor’s actions by computing a value function. This dual structure allows actor–critic methods to be more stable and converge faster than policy-gradient methods alone, as the critic’s evaluation stabilizes the updates provided to the actor. Modern variations, such as the asynchronous advantage actor–critic (A3C) [145], further refine this approach by decoupling the policy and value updates, thereby enabling more efficient learning processes and robust policy formulation in complex environments. This framework effectively addresses the high variance issue of policy-gradient methods while maintaining a continuous learning update, making it particularly suited for problems with high-dimensional action spaces and environments with stochastic dynamics.

## Training deep learning models

### Deep learning frameworks

For the development of deep learning models, several frameworks have gained prominence. The most widely used frameworks among academic researchers and industrial developers are PyTorch and TensorFlow, which include the high-level API Keras. These were primarily built for the Python programming language. Frameworks developed in other programming languages include MATLAB’s Deep Learning Toolbox, Java’s Deeplearning4j, and Caffe for C/C++. Specifically for medical applications, the Medical Open Network for Artificial Intelligence (MONAI)^1^, built on top of PyTorch, offers an extensive array of networks for various applications. These networks are not only ready to use but also easily modifiable to suit specific requirements.

**Fig. 17:**
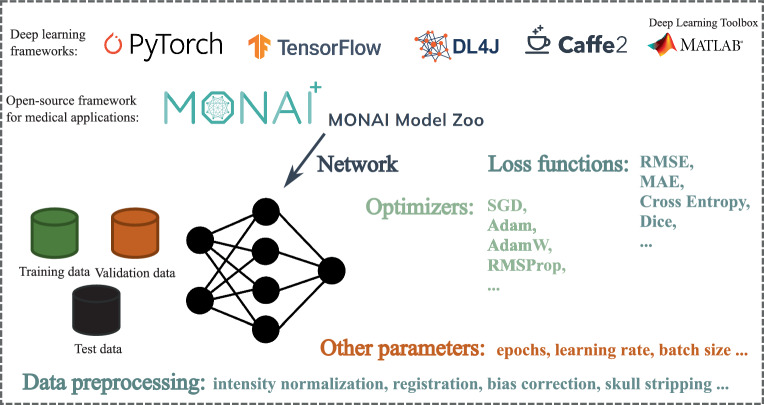
Many existing deep learning frameworks facilitate a plug-and-play approach, enabling researchers to efficiently train and implement deep learning models with greater ease and flexibility

### Data preprocessing

Converting the intensity of imaging data into a range of $[-1,1]$ (normalization) or $[0,1]$ (standardization) is a common data preprocessing step for all deep learning algorithms. It provides several advantages for both training and testing:

- Avoids saturation: many activation functions like sigmoid or tanh get saturated for very small or very large input values. In the saturated regions, gradients are near zero, leading to the vanishing gradient problem, which slows down or even halts training. By normalizing inputs to a range where the activation functions operate in their sensitive region, we can ensure that gradients are substantial enough for training to progress.
- Feature uniformity: normalizing ensures that all input features are on the same scale. Without normalization, features with larger scales might dominate the training process, making the model less sensitive to features with smaller scales. This is especially important for multimodality imaging data, since CT values range within a large scale of $[-1000,3000]$ HU, while MRI values are not universally standardized like CT values.
- Improved generalization: by ensuring that the model is trained on a standardized scale of input data, it is more likely that the model will generalize well to new, unseen data, provided the unseen data are also normalized in the same manner.

For MRI, bias correction, such as using the N4ITK method [146] to address bias field inhomogeneity, is an essential preprocessing step. It ensures consistent image quality and facilitates better feature extraction by the neural network. For example, in brain metastasis segmentation [12], false-positive segmentations might arise because of the hyperintensity in inhomogeneous regions. In addition to intensity normalization, the spatial normalization achieved by image registration is another important preprocessing step. Aligning multiple MRI scans or sequences (either intra- or interpatient) to a common spatial template, e.g., the Montreal Neurological Institute (MNI) space for brain imaging [147], is crucial for multimodal analyses and longitudinal studies. In addition, for brain imaging, skull stripping [148] is a preferred step to remove non-brain structures, which focuses the model’s attention on relevant brain tissues and helps to ensure data privacy.

### Loss functions

Mean squared error (MSE; i.e., the L2 loss) and mean absolute error (MAE; i.e., the L1 loss) are commonly employed when aiming to regress continuous values, such as predicting the radiation dose–response behavior of tissues. Huber loss offers a compromise between MSE and MAE, proving beneficial in scenarios with occasional outliers. Perceptual loss using VGG network features [149] is also commonly used for medical imaging to enhance high-level details.

For segmentation tasks, where delineating tumors and organs at risk is paramount, Dice loss or Jaccard/intersection over union (IoU) loss are often preferred in addition to cross-entropy loss. When categorizing patient responses or outcomes, cross-entropy loss is a go-to choice, especially for multiclass classification. A loss odyssey for medical image segmentation is available in [150].

Survival prognosis is a common task in radiation oncology. Cox proportional hazards models [151] are a class of effective survival models that typically use the negative partial likelihood loss function [152]. The partial likelihood loss only includes individuals who have experienced the event (i.e., uncensored data) at each observed event time. For each event time, it compares the risk set (those who have neither had the event nor been censored) to the risk of the individuals who experienced the event at that time. The partial likelihood method effectively handles right-censored data, since it considers only those individuals who have experienced the event of interest at each observed event time and compares their risk relative to others in the risk set at that time. The objective is to maximize this partial likelihood, which is equivalent to minimizing the negative log of this likelihood. For categorical survival prediction (i.e., survival or risk classification; for example, binary high-/low-risk classification), the cross-entropy loss [153] has been widely used. However, the regular cross-entropy loss may lead to a high prediction error and a heavy bias. Therefore, the negative log-likelihood function of a discrete time-to-event model has been proposed as a theoretically sound and easy-to-implement solution [153]. In many applications, other loss functions are commonly used as a joint loss together with the negative log-likelihood loss, for example, the ranking loss in the DeepHit method [154] and the MSE/MAE loss for normally distributed errors in the accelerated failure time model [155].

For medical data, class imbalance is a pervasive challenge that can skew the learning process and result in suboptimal model performance. Loss functions play a critical role in addressing this imbalance. Traditional loss functions like cross-entropy loss can be adapted using weighted or cost-sensitive learning, where classes are assigned different weights based on their representation [150]. The focal loss [156] is designed explicitly for scenarios where the foreground–background class imbalance is high, as it modulates the contribution of each sample to the loss based on its difficulty. Sensitivity-specificity losses [12, 157] have been demonstrated to be effective for brain metastasis segmentation, where the class imbalance problem is addressed by weighting sensitivity higher. Other techniques like oversampling, undersampling, and synthetic sample generation can be combined with these loss functions to provide a holistic approach to managing class imbalance. For example, the weighted random sampler from PyTorch is an effective choice.

Given the multifaceted challenges in radiation oncology, it is not uncommon to see hybrid or combined loss functions, which amalgamate the strengths of individual loss metrics to address complex objectives.

### Optimizers

Given the loss function, deep learning frameworks like PyTorch and TensorFlow will calculate the gradients of the loss function with respect to the trainable network weights automatically through the backpropagation mechanism [66, 158], and gradient descent optimization algorithms can be applied to update and train the network weights. Traditional optimizers like stochastic gradient descent (SGD) [158], revered for its simplicity and predictability, can sometimes be slow to converge and susceptible to local minima. Advanced variants like Momentum or Nesterov accelerated gradient descent enhance SGD by adding velocity components, thus alleviating some of its challenges [11]. Adaptive moment estimation (Adam) [159] has gained significant traction due to its adaptive learning rates for each parameter, making it robust against a range of initial settings and often leading to faster convergence. Its variant, AdamW [160], which incorporates weight decay, further refines the optimization landscape, potentially improving generalization in radiation oncology tasks. RMSProp [11], which adjusts the learning rate using a moving average of recent gradients, can also be effective in scenarios with noisy data. The choice among these optimizers should be influenced by the nature of the data, the architecture of the neural network, and the specific clinical objectives.

A comprehensive overview of gradient descent optimizers can be found in [11]. A visual explanation with 3D animations of different optimizers can be found https://towardsdatascience.com/a-visual-explanation-of-gradient-descent-methods-momentum-adagrad-rmsprop-adam-f898b102325c [161]. For most applications in radiation oncology, the Adam optimizer is recommended.

### Learning rate

The learning rate directly influences the convergence speed, stability, and eventual performance of a model [162]. Essentially dictating the step size in the optimization landscape, a high learning rate can expedite convergence but may overshoot minima or even diverge, while a very low learning rate ensures stable convergence but risks getting trapped in local minima or taking prohibitively long to train.

To address these challenges, learning rate schedulers, such as step decay, exponential decay, and cosine annealing, have been introduced to dynamically adjust the learning rate based on epochs or iterations. Cyclical learning rates, which vary the rate between two bounds, can also help in navigating the loss landscape effectively.

The influence of learning rate and its scheduling also varies with the choice of optimizer. For instance, SGD [158] often requires careful initial learning rate tuning and can greatly benefit from the aforementioned schedulers. Adam [159], which incorporates adaptive learning rates for each parameter, offers more robustness to initial choices but is not immune to benefits from dynamic rate adjustments. A fixed learning rate between $10^{-3}$ and $10^{-5}$ is commonly recommended for Adam. In essence, while modern optimizers and schedulers provide tools to alleviate some sensitivities associated with learning rates, their optimal determination and adjustment remain a blend of empirical experimentation and domain knowledge.

### Batch size

Batch size significantly influences both the model’s convergence behavior and its computational efficiency [162]. A smaller batch size offers more frequent weight updates, potentially leading to faster convergence and enabling the model to escape from local minima or saddle points, albeit at the cost of increased noise in the gradient estimates. Conversely, a larger batch size provides more accurate gradient estimates due to averaging across more samples, resulting in stable convergence but with a heightened risk of settling in sharp minima, which may impact generalization. Additionally, larger batches exploit the parallel processing capabilities of modern GPUs more effectively, leading to faster epoch times. However, they also demand greater GPU memory resources.

In summary, while smaller batches often promote better generalization and model robustness, larger batches accelerate training and offer computational advantages.

### Monitoring of overfitting

**Fig. 18:**
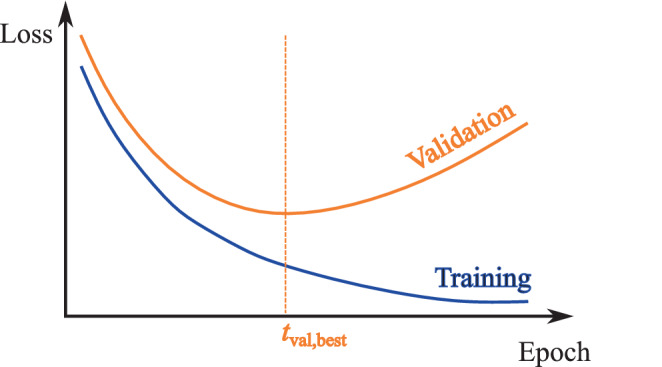
Plot of typical training and validation losses, where the best model is obtained at $t_{ \textrm{val,best}}$

Monitoring overfitting during the training of neural networks is of paramount importance to ensure that models generalize well to unseen data rather than merely memorizing the training dataset. Overfitting manifests when a model exhibits significantly better performance on training data compared to validation or test data. To detect this, it is common practice to split the dataset into separate training and validation subsets and closely observe the model’s performance metrics on both. Visualizing the learning curves, where training and validation losses are plotted against epochs, as displayed in Fig. 18, can provide clear indications of overfitting when the validation performance begins to degrade while the training performance continues to improve. With such monitoring, early stopping can be applied to mitigate overfitting. In other words, training will be stopped when the performance on the validation set starts to degrade (i.e., when the validation loss begins to increase), even if the training loss continues to decrease.

In addition, regularization techniques and dropout layers [163] can be employed to mitigate overfitting. Regularization methods add a penalty to the loss function to discourage overly complex models. Common techniques include L1 and L2 regularization. L1 regularization adds a penalty equivalent to the absolute value of the weights, which encourages the model to have more parameters whose values are close to zero. L2 regularization adds a penalty equal to the square of the magnitude of the coefficients, effectively penalizing large weights to prevent them from having too much influence. Dropout [163] is a technique used specifically in neural networks. It works by randomly setting a fraction of input units to zero at each update during training time, which helps to prevent neurons from co-adapting too much. This randomness forces the network to learn more robust features that are useful in conjunction with many different random subsets of the other neurons.

## Conclusion

This paper provides a comprehensive overview of deep learning principles tailored toward radiation oncologists and medical physics experts. It elucidates the fundamental principles of major deep learning models, encompassing MLPs, CNNs, RNNs, transformers, GANs, diffusion-based generative models, and reinforcement learning. For each category, it presents representative networks alongside their specific applications in radiation oncology. Moreover, it outlines critical factors essential for training deep learning models, such as data preprocessing, loss functions, optimizers, and other pivotal training parameters including learning rate and batch size. This manuscript is designed to improve the comprehension of AI-based research and software tools in radiation oncology. By doing so, it seeks to connect the intricate technological theories of AI with the practical aspects of clinical practice in radiation oncology.

## Appendix

### List of symbols and terms

**Table:**

Scalar value	Non-bold variable like $x$
Vector	Bold, lowercase variable like $\boldsymbol{x}$
Matrix	Bold, uppercase variable like $\boldsymbol{X}$
Step function	$\delta(x)=( \textrm{sign}(x)+1)/2$
Sigmoid function	$\delta(x)=\frac{1}{1+e^{-x}}$
tanh function	$\delta(x)=\frac{e^{x}-e^{-x}}{e^{x}+e^{-x}}$
Rectified linear unit (ReLU)	$\delta(x)=\max(0,x)$
Leaky ReLU	$\delta(x)=\max(x,\alpha x)$, e.g., $\alpha=0.01$
Swish function	$\delta(x)=x\cdot\frac{1}{1+e^{-\beta x}}$ where $\beta$ is trained
Softmax	$\delta(\boldsymbol{x})=\frac{e^{x_{i}}}{\sum_{j=0}^{K-1}e^{z_{j}}} \textrm{ for }i=0,{\ldots},K-1, \textrm{ and }\boldsymbol{x}=(x_{1},x_{2},{\ldots},x_{k-1})$
Concatenation	Stack feature maps along the channel dimension
Max pooling	Downsampling feature maps in a network using the maximum value in a local (typically $2\times 2$) neighborhood
Mean pooling	Downsampling feature maps in a network using the mean value in a local (typically $2\times 2$) neighborhood
$\mathbb{E}$	Mathematical expectation, i.e., mean
$\boldsymbol{I}$	Identity matrix
$\prod_{i=0}^{n}x_{i}$	product operation $\prod_{i=0}^{n}x_{i}=x_{0}\cdot x_{1}\cdot x_{2}\cdot\ldots\cdot x_{n}$
$\mathcal{N}(\boldsymbol{\mu},\boldsymbol{\Sigma})$	Gaussian distribution with mean vector $\boldsymbol{\mu}$ and variance matrix $\boldsymbol{\Sigma}$
Loss function	Measures the difference between a model’s predictions and labels
Mean absolute error (MAE) loss, i.e., L1 loss	$L1(\text{loss})=\sum_{i=1}^{n}|y_{i}-\hat{y}_{i}|/n$
Mean square error (MSE) loss, i.e., L2 loss	$L2(\text{loss})=\sum_{i=1}^{n}(y_{i}-\hat{y}_{i})^{2}/n$
Epoch	A full pass through the entire training dataset
Batch	A small subset of training data used to update model weights at each iteration
Batch normalization	A technique used in neural networks that normalizes the inputs of each layer using the mean and variance of the values in the current batch, thereby improving training speed and stability by reducing internal covariate shift
Learning rate	Step size for updating model parameters
Token	A piece of text that is treated as a single unit for processing
$\nabla$	Vector differential operator $\nabla\boldsymbol{f}=\left(\frac{\partial\boldsymbol{f}}{\partial x},\frac{\partial\boldsymbol{f}}{\partial y}\right)$
Score function	$\nabla_{\boldsymbol{x}}\log p_{t}(\boldsymbol{x})$, the direction of data flow to the highest probability
Q‑value $Q(s,a)$	The maximum expected reward an agent can reach in reinforcement learning by taking a given action $a$ from the current state $s$

## Footnotes

1 https://monai.io/.
